# Advancement in wrist worn physical activity trackers: technological developments and measurement accuracy: a systematic review

**DOI:** 10.3389/fdgth.2026.1820224

**Published:** 2026-08-21

**Authors:** Tristan Hogue, Alexia Khoury, Pressila Njeim, Nicole Aswad, Layale Youssef, Sarah Birtulescu, Andréa Faust, Guy Hajj-Boutros

**Affiliations:** 1 Research Institute of McGill University Health Centre, McGill University, Montréal, QC, Canada; 2 Centre de Recherche de l’Institut Universitaire de Gériatrie de Montréal, Montreal, QC, Canada; 3 Department of Surgery, Vanderbilt University Medical Center, Nashville, TN, United States

**Keywords:** accelerometry, accuracy, energy expenditure, heart rate, physical activity, PPG, step count, validation studies

## Abstract

**Background:**

Wrist-worn physical activity trackers have advanced rapidly over the past two decades, evolving from simple accelerometer-based devices to multi-sensor systems integrating photoplethysmography, advanced movement classification, and machine-learning algorithms. Despite widespread use in both research and clinical settings, concerns remain regarding the accuracy and reliability of key metrics such as heart rate, energy expenditure, step count, and activity intensity.

**Methods:**

This systematic review followed PRISMA guidelines (PROSPERO: CRD42024551740). MEDLINE, Scopus, PubMed, and SPORTDiscus were searched for studies evaluating the accuracy or acceptability of wrist-worn activity trackers in laboratory or free-living-simulated conditions. Eligible studies included healthy or general-population samples using any wrist-worn consumer or research-grade device. Reference standards included ECG, indirect calorimetry, doubly labelled water, manual step counting, and research-grade accelerometry. Of 1,659 records identified, 47 studies met inclusion criteria.

**Results and discussion:**

Accuracy improved substantially over time, although variability across brands, activities, and populations persisted. Early heart-rate monitors (pre-2010) frequently reported Pearson correlation coefficient as low as 0.50 during exercise, while still being under the validity standard (0.90) at rest. Modern devices such as Apple Watch and Garm*in vivo*smart demonstrate markedly increased validity scores (typically above 0.80 during exercise), reflecting improvements in optical sensor quality and motion-artifact filtering. Energy-expenditure accuracy showed the least improvement; even recent devices exhibit 10%–40% error relative to indirect calorimetry, reflecting the inherent challenge of estimating metabolic rate without direct O₂ and CO₂ measurement. Step-count accuracy, prior to 2017 showed errors of up to 25%, now commonly falls below 5% for steady-state walking due to improved accelerometer precision and gait-pattern recognition. Classification of physical-activity intensity has also improved, with misclassification decreasing from >20% in early models to 10%–15% in current multi-sensor devices. Acceptability was generally high across brands, with long wearing time and good participant satisfaction.

## Introduction

Over the past two decades, wrist-worn physical activity trackers have transformed how individuals and researchers monitor energy expenditure (EE), heart rate (HR), step count, and physical activity intensity. These devices leverage accelerometry, photoplethysmography (PPG), and advanced machine-learning algorithms to provide real-time feedback on health-related metrics, making them essential tools in clinical settings, fitness monitoring, and epidemiological research (1). While their convenience and accessibility have driven widespread adoption, concerns regarding measurement accuracy and reliability persist, particularly when compared to gold-standard methods such as indirect calorimetry, electrocardiography (ECG), and metabolic carts (2–4).

The evolution of wrist-worn devices has been characterized by substantial improvements in sensor technology, algorithm sophistication, and integration with smartphone applications and cloud-based analytics. Early-generation devices, such as pedometers and basic accelerometers, provided crude estimations of step count and activity levels; however, modern smartwatches now incorporate multi-sensor fusion technologies capable of estimating oxygen consumption (VO_2_), heart rate variability (HRV), and metabolic equivalents (METs) (5, 6). Despite these advancements, measurement accuracy remains variable across different activities, exercise intensities, and user demographics, highlighting the need for systematic validation studies (7–9). Several studies have reported discrepancies between consumer-grade wrist-worn devices and criterion measures, with energy expenditure estimates exhibiting the largest error margins (6, 10). Heart rate tracking is generally more reliable but remains sensitive to factors such as skin tone, wrist placement, and motion artifacts (7, 11). Step count accuracy is typically high during steady-state walking but decreases during non-ambulatory activities, including cycling and resistance training (12, 13). These inconsistencies underscore the importance of validating devices across diverse populations, activities, and exercise intensities (14).

### Rationale for a systematic review

Given the rapid technological advancements in wrist-worn activity trackers and their increasing use in research and healthcare settings, a comprehensive systematic review is warranted to assess the accuracy of these devices in measuring energy expenditure, step count, heart rate, and activity intensity, as well as to examine technological developments over time and evaluate their impact on measurement precision. This review aims to provide a critical synthesis of the existing literature, offering insight into the strengths and limitations of wrist-worn physical activity trackers while guiding future research directions and industry advancements.

## Methods

This systematic review was conducted in accordance with the Preferred Reporting Items for Systematic Reviews and Meta-analyses (PRISMA) guidelines. The review protocol was registered in the International Prospective Register of Systematic Reviews (PROSPERO; registration number: CRD42024551740).

### Search strategy

A comprehensive literature search was conducted in MEDLINE, PubMed, Scopus, and SPORTDiscus to identify studies evaluating the accuracy, validity, reliability, or acceptability of wrist-worn physical activity trackers. The search strategy combined terms related to wearable devices, wrist placement, physical activity tracking, and measurement accuracy. Search terms were adapted for each database using database-specific syntax, Boolean operators, truncation, and controlled vocabulary where applicable.

Briefly, the search combined terms for wearable technologies, such as “wearable device,” “wearable technology,” “fitness tracker,” “activity tracker,” “smartwatch,” “wrist-worn,” and “accelerometer,” with terms related to measurement outcomes and validation, including “accuracy,” “validity,” “reliability,” “reproducibility,” “measurement error,” “heart rate,” “energy expenditure,” “step count,” “physical activity,” and “activity intensity.” No restrictions were placed on sex or age. In addition, a snowball search was performed by screening the reference lists of relevant articles and previous systematic reviews identified through the database search.

### Selection of studies

This review examined both research-grade devices (activity trackers available exclusively for research purposes) and commercial devices (those available to the general public). The included studies were limited to the community-based everyday-life setting. Laboratory-based studies were included provided that they replicated everyday conditions, while studies involving institutionalized or hospitalized patients were excluded. No restrictions were imposed on the duration of observation in the included studies.

The inclusion criteria were indicated as follows: (1) studies involving the general population, with no restrictions on sex or age; (2) use of a wrist-worn wearable activity tracker; and (3) reporting at least one quantitative measure of acceptability, including wearing time, data availability, or questionnaires to assess acceptability. The exclusion criteria were indicated as followed: (1) device not worn on the wrist; (2) studies measuring sleep; (3) studies on patients who were institutionalized or hospitalized.

For the accuracy objective, the population of interest was the general population, the index test was required to be a wrist-wearable activity tracker, and the reference standard could include another device or any method used to assess physical activity, including questionnaires or direct observation. Outcomes of interest could be any measure of physical activity, including but not limited to energy expenditure, step count, heart rate, distance, speed, activity count, activity time, and intensity of physical activity.

All studies reporting primary data were considered eligible for inclusion, with the exception of case reports and case series.

Using the predefined inclusion and exclusion criteria and a piloted screening form, titles and abstracts of the retrieved articles were first screened. Full texts of studies deemed potentially eligible at this stage were then reviewed to determine final inclusion.

### Data extraction

Data were extracted into Microsoft Excel for Microsoft 365 (Microsoft Corporation, Redmond, WA, USA). Extracted information included general study information (first author, publication year, study type: prospective vs. retrospective and observational vs. interventional, follow-up duration in days, and setting: laboratory vs. field), population characteristics (number of participants, underlying health condition such as healthy, severe obesity, or chronic joint pain, gender, and age distribution as mean ± SD, median with range, or first and third quartiles), measures of accuracy (step count, distance, speed, heart rate, activity count, time spent being active, and physical activity intensity), and device acceptability, including data availability, wearing time, and ease of use.

### Diagnostic accuracy measures

When available, the mean absolute percentage error (MAPE) or the mean percentage error was extracted. If these were not available, other measures were extracted in the following priority order: mean difference, mean bias (Bland-Altman), accuracy determined through intraclass correlation coefficient (ICC), and correlation coefficient (Pearson or Spearman). For outcomes that were dichotomized with calculated sensitivity and specificity, these values were reported. When available, measures of variability or 95% CIs were included for all available measures. For biometric outcomes reported with varied reporting metrics, data presentation was standardized to enhance clarity and comparability. When multiple error metrics were reported (e.g., bias and ICC), the most commonly used statistic across reviews was selected to maintain consistency. To avoid double-counting, primary studies reported within the included systematic reviews were cross-checked, and duplicate records were removed so that each original study contributed only once to the synthesis.

### Synthesis of results

Due to substantial heterogeneity across studies, including variability in participant characteristics, settings (laboratory vs. free-living), device generations, sensor modalities, reference standards, and the statistical metrics used to quantify accuracy, a quantitative meta-analysis was not conducted. Instead, a narrative synthesis is provided, structured by outcome domain and by device type. For the accuracy objective, results were synthesized only for devices evaluated in at least two independent studies using comparable reference standards. These devices include major brands such as Apple, Garmin, Fitbit, Polar, WHOOP, COROS, and selected research-grade devices.

### Ethics approval

This systematic review was based on published data and therefore did not require approval from a research ethics board.

## Results

### Overview

**Figure 1:**
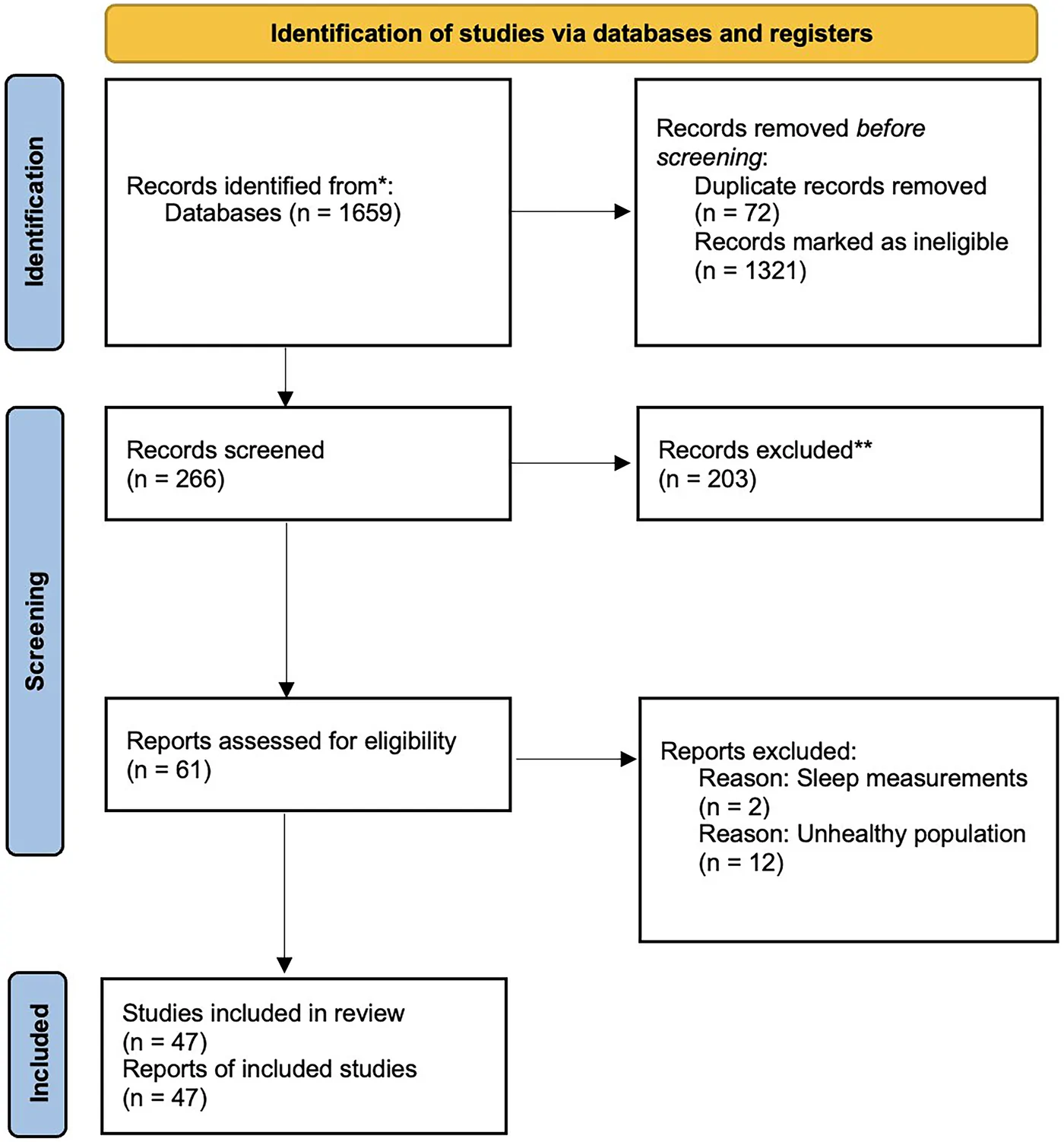
Flow diagram nof the article screening and selection steps.

The search identified 1,659 records of studies using wrist worn devices, of which 47 studies met the eligibility criteria and were included in the systematic review. The study flow diagram is presented in Figure 1. Characteristics of the included studies are summarized in Supplementary Table S1 for the accuracy and acceptability objectives.

Studies included studies were published between 2002 and 2025, evaluating the accuracy and validity of wrist-worn devices. The included studies employed diverse methodological designs, including validation against gold-standard measures such as ECG, indirect calorimetry, and observational methods. Sample sizes ranged from small laboratory cohorts of 4–20 participants to larger observational studies with over 170 subjects, as well as one cross-sectional study with a sample size of 1. The devices assessed included popular commercial brands such as Fitbit, Apple Watch, Garmin, Polar, Xiaomi, and WHOOP.

Considerable heterogeneity was observed in study design, activity protocols, and reference standards, precluding quantitative pooling. Therefore, a narrative synthesis was conducted for each outcome of interest, including heart rate, energy expenditure, step count, and physical activity intensity. Overall, the findings reveal significant improvements over time, with earlier studies (pre-2010) reporting higher measurement errors, whereas recent investigations demonstrate marked progress attributable to sensor advances, multi-sensor fusion, and machine learning algorithms.

### Accuracy

#### Heart rate

Early validation studies from the 2000s reported substantial errors in wrist-based heart rate monitors, with correlations as low as 0.50, and devices like the Instapulse heart rate monitor 100 failing to meet the validity standard (0.90) across all conditions, including rest (15).

Between 2009 and 2015, improvements in sensor technology led to enhanced accuracy. Porto et al. (16)(2009) found that the Polar S810 showed excellent agreement with ECG in measuring short-term heart rate variability, with no significant differences in variability metrics across postures, indicating reliability for clinical and research purposes (16). Similarly, El-Amrawy et al. (17) reported that the Apple Watch achieved the highest accuracy at 99.9%, while the Motorola Moto 360 had the lowest at 92.8%, with precision ranging from 5.9% to 20.6% (17). Heart rate correlation coefficients ranged from 0.67 (Mio Alpha) to 0.95 (Apple Watch), with percentage errors between 1% and 9% across four devices (18).

From 2017 onwards, newer devices demonstrated further improvements. Dooley et al. (14) reported that the Apple Watch had the lowest mean absolute percentage error (MAPE) of 1.14% to 6.70%, with higher errors observed for Garmin and Fitbit devices (14). Bai et al. (2) found that the Fitbit Charge HR was most accurate during sedentary periods (MAPE 7.2%), and the Apple Watch performed better during aerobic activities (2). Xie et al. (19) indicated that the Samsung Gear S3 had the highest variation across physical activity conditions (MAPE 34%) but performed well at rest (MAPE 4%) (19).

From 2019 onwards, research focused on validating high-end and medical-grade devices. Gilgen-Ammann et al. (20) demonstrated that the Polar Vintage maintained high signal quality (99.6%) and excellent agreement with ECG (*r* = 0.997), making it a gold standard for HRV measurement during intense activity (20).

More recently, Hajj-Boutros et al. (4) reported that the Apple Watch achieved errors below 5%, with ICCs close to 1.00 during treadmill exercise, indicating excellent validity (4). Giordano et al. (21) showed that heart rate extracted from a wrist-worn ultrasound-based IoT device had a very high correlation with ECG (*r* = 0.99) (21). Similarly, Lopez-Ortiz et al. (22) found no significant differences in mean HR between the KCO8 smartwatch and a cuff-based reference, with strong correlation and excellent reliability (ICC ≈ 0.898) (22). During high-intensity and high-intensity interval trainings protocols, device accuracy declined, with some devices experiencing data loss and increased bias, especially at heart rates above 160 bpm (23).

Overall, the evolution from early 2000s devices to recent high-end monitors shows substantial improvements, with current research-grade devices demonstrating errors often below 5%, high correlations, and ICCs exceeding 0.95, though accuracy can decline during high-intensity exercise and rapid heart rate changes (15, 16, 20, 23).

#### Energy expenditure

Energy expenditure (EE) remains the most challenging physiological variable for wrist-worn wearables to measure accuracy. Unlike heart rate or steps, EE is not directly measured but estimated using algorithms that integrate accelerometry, user characteristics, and, in some cases optical HR data (10, 14). In contrast, criterion methods such as indirect calorimetry require precise measurement of oxygen consumption (VO_2_) and carbon dioxide production (VCO_2_), while doubly labeled water captures whole-body CO_2_ turnover in free-living conditions. Because commercial wearables do not directly measure gas exchange or substrate oxidation, a substantial estimation error is almost inevitable.

Early validation studies from the 2010s reported that wrist-worn devices such as the Polar Activity Watch 200 showed limitations in accurately estimating energy expenditure (EE), particularly during longer activities, often underestimating EE compared to criterion methods like the Oxycon Mobil (24). During this period, errors ranged from about 9% to over 40%, with devices like Mio ALPHA exhibiting high variability in estimates (18).

Between 2017 and 2019, several studies indicated that consumer-grade devices generally underestimated EE during various activities. Roos et al. (8) found errors during aerobic running ranged from −25% to +38%, with the Polar V800 being relatively more accurate (8). Dooley et al. (14) reported that the Apple Watch overestimated EE with a mean absolute percentage error (MAPE) of up to 210%, while other devices like Fitbit and Garmin showed large errors during baseline and moderate intensities (14).

In subsequent years, improvements in device algorithms yielded better estimates. Bai et al. (2)(2018) demonstrated that the Apple Watch 1 had a MAPE of 15.2%, with stronger validity during aerobic activities compared to Fitbit Charge HR (2). Zhang et al. (5) showed that the Apple Watch had good agreement with indirect calorimetry during treadmill walking at speeds around 160–188 m/min, with errors around 10%–12%, supporting its use in controlled settings (5).

From 2020 onwards, newer studies indicated further progress. Heinz et al. (25) reported that the Apple Watch Series 7 tended to underestimate EE by about 27 kcal on average, but maintained a high correlation with spirometric calorimetry (*r* = 0.93) (25). Similarly, Zhao et al. (26) found that the ActiGraph accelerometers demonstrated the highest accuracy with an average MAPE of 14.51%, although many devices still tended to underestimate EE, especially at higher intensities, with errors ranging from 16.6% to 42.8% (26).

Overall, while early studies showed significant inaccuracies and underestimation, recent research indicates that high-end devices like the Apple Watch and ActiGraph have improved accuracy, with errors often below 15%, though some devices still exhibit limitations, especially during high-intensity or prolonged activities (18, 24, 26).

#### Step count

Early-generation wrist-worn devices from the 2000s relied on basic uni-axial accelerometers and simple threshold-based step-detection algorithms, resulting in substantial errors, particularly outside of steady-state walking.

Early studies from the 2010s reported that wrist-worn devices such as the Fitbit Zip and Yamax Digi-Walker demonstrated high correlation and reliability in step counting, with no significant differences compared to reference devices, although some devices, like Fitbit, tended to measure slightly more steps per day (27). Takacs et al. (28) confirmed that the Fitbit One showed high accuracy during walking, with less than 1.3% error, but its distance measurements were less accurate (28).

Between 2015 and 2017, several studies highlighted that most wrist-worn activity trackers tended to underestimate steps, especially at lower speeds or outdoor walking, with errors ranging from less than 2% to over 26%. Storm et al. (12) found that devices like Movemonitor and ActiGraph had minimal errors (<2%) and performed well in step counting, but some consumer devices overestimated step counts significantly, with Nike + Fuelband overestimating by over 250 steps on average (12).

In 2018, research indicated that the accuracy of wrist-worn step counters varied across devices and conditions. Bai et al. (2) reported that the Apple Watch 1 and Fitbit Charge HR accurately tracked steps during aerobic activity, with errors below 10% (2). Veerbhadrappa et al. (29) confirmed that the Apple Watch demonstrated very high accuracy, with a total error of only 0.034% (∼1 step), and slight overestimations at various walking paces (29).

More recent studies from 2019 onwards showed that the Apple Watch consistently demonstrated high precision in step counting, with Breteler et al. (30) reporting a MAD of 7.7%, comparable to the ActiGraph, while Yamax Digiwalker had higher variability with a MAD of 20.3% (9). Xie et al. (19) found that step measurement accuracy remained high across devices, with the Dongdong pedometer showing very low variation (MAPE 1%), and the Apple Watch 2 exhibiting higher variation (MAPE 42%) (30).

Overall, from early validation efforts to recent research, wrist-worn devices such as the Apple Watch, Fitbit, and others have demonstrated increasingly accurate and reliable step counting, with errors often below 10%, although some devices and conditions can lead to variability and over- or underestimation (9, 27, 29).

#### Physical activity

The classification of physical activity intensity has historically been one of the most challenging tasks for wrist-worn devices. Prior to 2010, most trackers relied solely on accelerometry to estimate activity intensity, resulting in misclassification errors often exceeding 25%–35% (31, 32). Light activities were frequently labeled as sedentary, and moderate-intensity movements were often underestimated. Without heart-rate integration, early devices struggled to accurately assess activities such as cycling, resistance training or lifestyle movements, where wrist acceleration did not correspond linearly with metabolic demand.

Early studies from 2012 demonstrated that wrist-worn devices such as the GENEA achieved high activity classification accuracy, with the right wrist data correctly classifying activities at 96.97% (*κ* = 0.954) and the left wrist at 95.93% (*κ* = 0.934). These results were confirmed by 10-fold cross-validation, which showed overall accuracy of 97.2% for the right wrist and 96.01% for the left wrist, indicating excellent classification performance (5).

In 2013, Mannini et al. reported that activity classification accuracy using 12.8-second windows reached 95.0% for ankle data, while wrist data accuracy was slightly lower at 84.7%. When shorter 4-second windows were used, wrist data accuracy decreased marginally to 84.2%, suggesting a small decline in performance with shorter time segments (32).

Furthermore, overall activity classification accuracy across 14 activities was approximately 53%, with high accuracy (>80%) for activities like basketball, jogging, and treadmill walking. However, classification accuracy varied depending on activity intensity, with sedentary activities around 70%, light and moderate activities near 45%–46%, and vigorous activities about 78%. Cycling activities, in particular, had low accuracy (<25%), likely influenced by activity type and device placement (31).

In studies from 2021 to 2025 included in the dataset, newer devices such as WHOOP Strap, and Fitbit Charge 4 demonstrated improved activity-intensity classification accuracy, with error margins generally falling below 10%–15% under mixed-activity conditions (33). Devices incorporating machine-learning-based recognition models performed particularly well; for example, wrist-based WHOOP and Polar units correctly classified activity bouts in more than 85% of monitored periods in several recent validation studies. Despite this progress, accuracy remains dependent on activity modality, with resistance training, high-intensity interval exercise and mixed-movement sport still presenting classification challenges. These discrepancies are reflected in the dataset, where wrist-based devices often underestimate high-intensity interruptions and overestimate continuous moderate-intensity activities with substantial arm movement.

Although physical-activity classification remains imperfect, accuracy has improved considerably in the past two decades. Errors exceeding 30% in early accelerometer-only devices have now narrowed to less than 10%–15% in many contemporary multi-sensor systems (31, 34). Continuous refinement of sensor fusion, contextual recognition algorithms, and individualized calibration models is expected to drive further improvements in the coming years.

## Discussion

**Figure 2:**
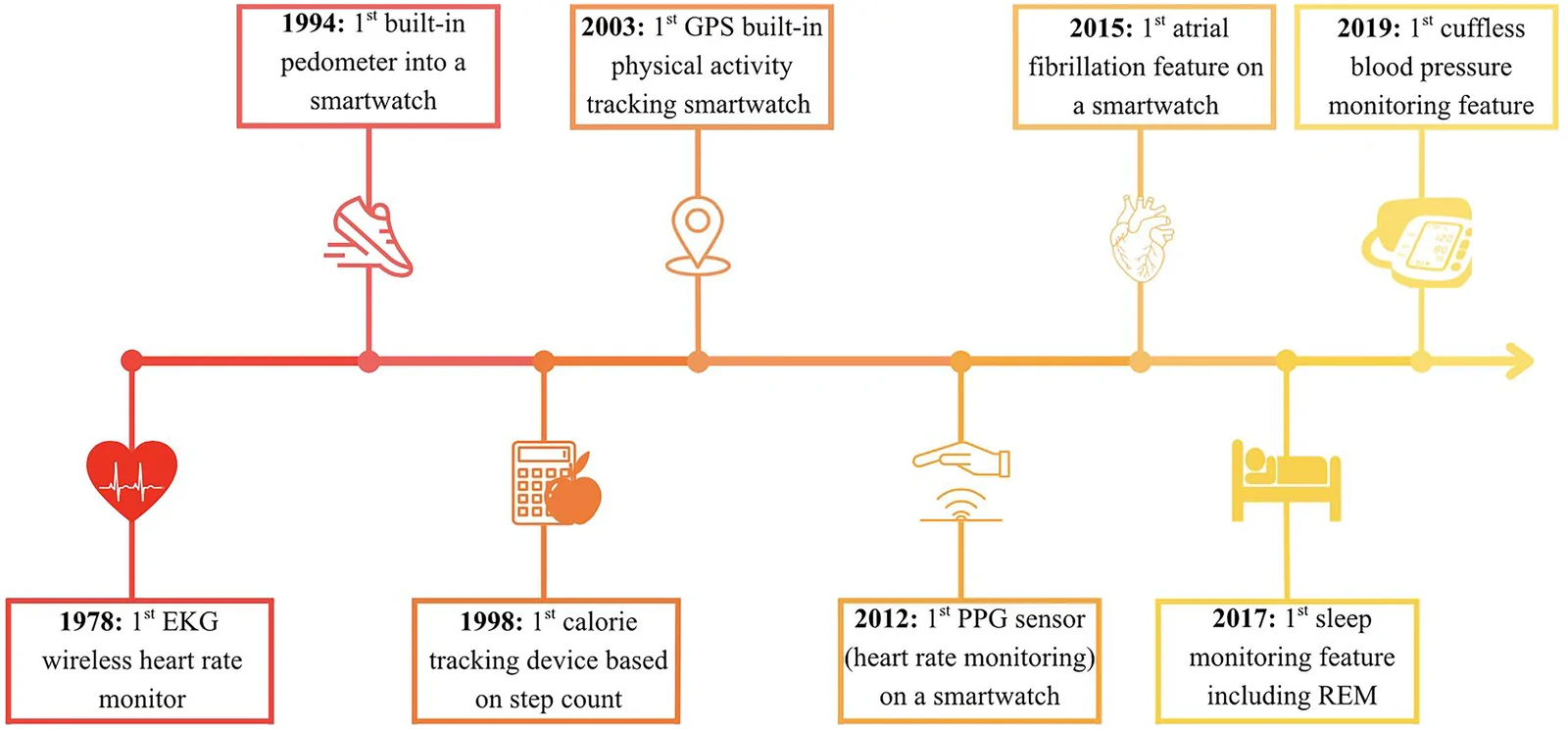
Timeline of key milestones in Wrist-Worn wearable health devices and activity monitoring.

**Figure 3:**
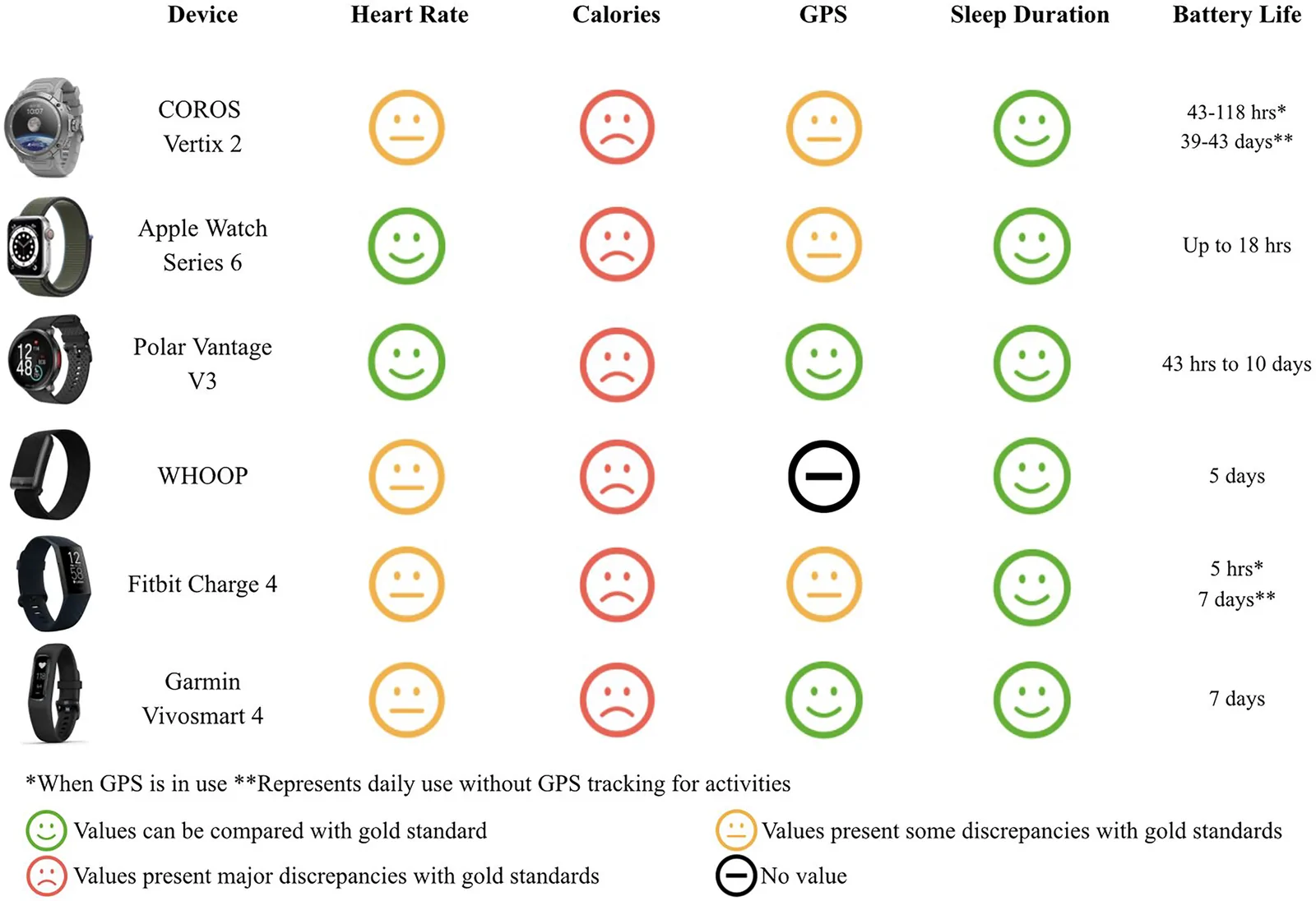
Comparative accuracy of Wrist-Worn wearables for heart rate, energy expenditure, GPS, and sleep duration, With reported battery life.

This systematic review examined the accuracy and acceptability of wrist-worn wearable devices over the past two decades. Overall, the evidence highlights substantial technological progress alongside persistent limitations. Across the 47 included studies, accuracy for heart rate, energy expenditure, step count, and physical activity estimation varied considerably by brand, device generation, activity type, and the reference method used. As shown in Figure 2, the field has progressed from early accelerometer-only devices in the 2000s to current multisensor systems, with clear improvements in measurement precision, particularly for heart rate and step count. However, challenges remain for more complex outcomes, especially energy expenditure and physical activity intensity. Figure 3 provides a comparative summary of the accuracy of widely used wrist-worn devices across key outcomes, including heart rate, energy expenditure, and sleep duration, and highlights where performance is consistently strong vs. where measurement error remains substantial.

### Evolution of accuracy across outcomes

Early validation studies conducted before 2010 consistently reported large errors across all metrics, especially for energy expenditure, indicating that early devices were not reliable for precise measurements (7, 18). Devices from Polar, Garmin, and Fitbit released after 2015 showed significant improvements, with several independent studies reporting lower mean absolute percentage errors during steady-state activities (14). This progress reflects advances in sensor fusion, incorporating multi-axis accelerometry, optical photoplethysmography, gyroscopes, and increasingly sophisticated proprietary algorithms. Research published between 2020 and 2025, including studies validating the Garm*in vivo*smart (35), Apple Watch (4), and WHOOP systems (34), consistently shows enhanced precision during controlled laboratory walking and running tasks. Despite these gains, free-living accuracy remains variable, highlighting the ongoing challenge of contextual variability for wearable sensor algorithms.

### Energy expenditure remains the most challenging metric

Among all outcomes, energy-expenditure estimation remains the least accurate across brands and device generations. Despite improvements in sensor technology and algorithm development, recent validation studies continue to report substantial errors compared with criterion methods, particularly in free-living settings and during higher-intensity activities (7, 10, 14, 26). This limitation stems from the inherent complexity of the physiological processes underlying caloric expenditure. Accurate estimation requires assessment of oxygen consumption (VO_2_) and carbon dioxide production (VCO_2_), achievable only through indirect calorimetry or doubly-labeled water (7, 10). In contrast, wrist-worn devices infer energy expenditure indirectly through proxies such as motion, wrist acceleration, user characteristics, wrist temperature, and heart rate, which limits accuracy across different activities and intensities (6, 14). Several studies included in this review confirm that despite algorithmic improvements, energy-expenditure prediction remains inherently constrained by the lack of direct gas-exchange measurement (4, 10, 14, 26). Consequently, the discrepancy between wearable-derived and calorimetry-derived EE values should be considered when interpreting results for clinical or research applications, particularly when precise metabolic assessment is required.

### Significant progress in heart rate tracking

In contrast, heart-rate measurement shows the clearest improvement over time. Early PPG-based systems were highly susceptible to motion artefact, skin-tone interference, and loose wrist contact (36). However, validation studies from 2021 to 2024 demonstrate that modern devices, such as the Garmin Fenix, Apple Watch, and Polar Vantage, achieve minimal mean absolute percentage errors under steady conditions. Several studies in the dataset [e.g., (4, 35, 37)] confirm that heart-rate accuracy is now sufficiently high for use in exercise monitoring and research, though accuracy deteriorates during high-intensity or arm-dominant activities (<5% variability). Nevertheless, contemporary wearables represent a considerable step forward compared with early 2000s devices.

### Improvement in step count and activity classification

Step-count accuracy has similarly benefited from advances in sensor sensitivity and machine-learning-based classification. Before 2010, step-count errors were significant, particularly at slow walking speeds (38). Studies conducted between 2020 and 2023, such as validations of Fitbit Charge 4 (35), Polar Elixir (2024 study), and Garm*in vivo*smart, show lower error rates in standardized conditions. Activity classification has also improved, although variability persists. Earlier-generation devices struggled with distinguishing moderate from vigorous activity and misclassified non-locomotor upper-body movements. Recent studies demonstrate improved detection of moderate-to-vigorous physical activity (MVPA), though misclassification rates remain in the range of quite high in some populations (39). Machine-learning models embedded in newer wearables appear more robust, but free-living environments continue to introduce variability.

### Strengths and limitations of the evidence base

The included studies differ widely in design, population characteristics, device types, reference standards, and outcome measures. Controlled laboratory protocols tend to yield more favorable accuracy estimates than free-living investigations, reflecting the influence of movement variability on sensor reliability. Some studies relied on small sample sizes, young or healthy adult populations, or short monitoring periods, which may limit generalizability to older adults or clinical groups. Another limitation is the rapid evolution of wearable technologies, whereby devices validated in 2020 may already be technologically outdated by 2025, complicating longitudinal comparisons of device accuracy. An important limitation of the current evidence base is that most validation studies were conducted in healthy adults under standardized laboratory conditions. This limits the generalizability of accuracy estimates to clinical populations, in whom movement patterns, gait speed, arm swing, tremor, assistive-device use, fatigue, and disease-related functional limitations may differ substantially from healthy individuals. These factors may reduce the accuracy of wrist-worn devices, particularly for step count, physical activity intensity, and energy-expenditure estimation, because many commercial algorithms are trained and validated primarily using movement signatures from healthy users. Therefore, device performance observed in healthy adults should not be assumed to apply directly to clinical populations without population-specific validation.

### Implications for research and practice

The findings of this review confirm that wrist-worn devices are now sufficiently accurate for many research and health-monitoring applications, particularly for heart rate and step count. However, caution is warranted when precise energy-expenditure measurement is required. For clinical trials or metabolic studies, indirect calorimetry or doubly-labeled water remains essential. Nevertheless, the observed improvements especially in heart rate and step count, is encouraging and suggest that wearables will continue to expand their role in health assessment, remote monitoring, and personalized exercise prescription.

### Future research and suggestions for biotech companies

Future research on wrist-worn activity trackers should prioritize methodological standardization and the development of unified validation protocols across laboratories and populations. One of the major limitations identified in the present review is the inconsistency in reference standards and outcome metrics, which complicates direct comparison between devices and across studies. Establishing consensus guidelines, similar to those used in cardiometabolic biomarker research, would allow more rigorous benchmarking of device accuracy, especially for complex outcomes such as energy expenditure. Research should also expand validation efforts to include understudied groups, such as older adults, individuals with chronic diseases, and people with higher BMI, who are often underrepresented despite being frequent users of wearables in both clinical and lifestyle contexts (40).

Several technological priorities emerge for biotech companies. First, improving energy-expenditure estimation remains a critical challenge. As demonstrated across the included studies, even the most advanced devices continue to show margins of error exceeding 10%–20% in free-living conditions due to the physiological complexity of EE measurement, which ideally requires accurate quantification of oxygen consumption and carbon-dioxide production (VO_2_ and VCO_2_) or direct measurement of heat production. Biotech companies should therefore explore hybrid sensor systems that move beyond accelerometry and heart rate alone, integrating additional physiological signals (e.g., skin temperature, respiration, galvanic skin response) and individualized metabolic models to approximate the underlying metabolic pathways more accurately.

Another important avenue is the refinement of photoplethysmography (PPG) technology. Although significant progress has been achieved since 2020, motion artefact remains a major barrier to accuracy during high-intensity or upper-body activities (36). Companies should invest in improved optical configurations, machine-learning-driven noise reduction, and adaptive sampling that responds dynamically to changes in movement intensity. Multi-wavelength PPG systems, already emerging in some newer models, may further enhance performance in users with darker skin tones or tattoos, conditions known to interfere with signal penetration (41, 42).

For clinical trials and healthcare applications, the central issue is not simply whether a device uses a proprietary or “black-box” algorithm, but whether there is sufficient evidence supporting the validity of that device and algorithm for the intended context of use. Accuracy should therefore be evaluated in relation to the target population, outcome of interest, activity conditions, and intended application (43). Greater transparency in data processing may support reproducibility and interpretation, but context-specific validation remains essential before wearable-derived outcomes are used for clinical decision-making or as endpoints in research studies. Finally, the next generation of wearable technology should incorporate adaptive, personalized algorithms that adjust to an individual's physiological profile over time. Longitudinal learning models trained on the user's historical heart rate, movement patterns, and daily rhythms could substantially reduce error and improve sensitivity to meaningful physiological changes, particularly in clinical populations undergoing lifestyle interventions or pharmacological treatment.

Overall, advancing the scientific and clinical utility of wrist-worn activity trackers will require close collaboration between academic researchers, clinical scientists, and biotech developers. By addressing current technological limitations, improving algorithm transparency, and expanding validation efforts across diverse populations, the field can move toward wearables that not only track activity, but provide reliable, clinically actionable data to support precision health and disease prevention.
