# Supplementary material for: Advancement in wrist worn physical activity trackers: technological developments and measurement accuracy: a systematic review
Source: Front Digit Health. 2026 Aug 21;8:1820224. doi: 10.3389/fdgth.2026.1820224 (PMC13542415; doi:10.3389/fdgth.2026.1820224)
Supplement: Supplementary file 1 [file Table1.pdf]

Authors and year	Study design and aim	Subjects	Measurement methods	Type of device tested	Variables	Results
**Terbizan et al. (2002) (15)**	Validation study<br><br>(Accuracy of different heart rate monitors vs ECG at rest and on a treadmill)	-n: 14 subjects<br>-All males<br>-Age: 19.6 yrs (2.3 SD)<br>-Healthy population	Electrocardiogram (one-channel, Burdick E-100 Electrocardiograph)	Chest straps: Polar Vantage XL, Polar Accurex II Accumen Basix Performance Accelerator, Cardiosport Exel ZW-8, Cardiochamp-Sensor Dynamics, Inc. The Cateye Model PL-6000, Instapulse HR monitor Model 100	Heart rate	-Four of the heart rate monitors – the Polar Vantage XL, Polar Accurex II, Cardiochamp, and Cateye-PL 6000 – demonstrated high accuracy during rest and moderate activity, with Pearson correlation coefficients $r \geq 0.90$ and a standard error of estimate SEE ≤ 5. They were found to be valid during rest and at the first two speeds of testing.<br>-Accumen Basix Performance Accelerator was valid only during the first two speeds of testing.<br>-Cardiosports Exel ZW-8 and the Instapulse heart rate monitor 100 failed to meet the validity standard of $r \geq 0.90$ during any experimental condition.<br>-Instapulse heart rate monitor 100 had the lowest Pearson correlation coefficient during exercice ($r = 0.50$).
**Leenders et al. (2003) (38)**	Experimental study<br><br>(Accuracy of activity monitors on treadmills)	-n: 28 subjects<br>-11 males, 17 females<br>-Age: 23.7 yr (3.9 SD)<br>-Healthy population	Indirect calorimetry	Device : Tritrac-R3D (TT), Computer Science & Applications, Inc. (CSA), and Mini-LoggerO (ML)	Energy expenditure, Measurement of bodily movement	-Tritrac, CSA, Yamax: quantify walking (light-moderate intensity)<br><br>-Activity monitors: correlate with energy expenditure (indirect calorimetry)
**SE Crouter et al. (2003) (44)**	Cross-sectional study<br><br>(Evaluation of Polar S410 heart rate monitor's accuracy in estimating exercise energy cost)	-n: 20<br>-10 males, 10 females<br>-Healthy population	Indirect calorimetry	Polar S410	VO2max and Hrmax – Energy Eexpenditure	-Males: No EE differences between AHRM, PHRM, and IC ($P > 0.05$)<br>-Females: PHRM overestimated EE on treadmill, cycle, and rower ($P < 0.05$)<br>-AHRM improved EE estimation for females but still overestimated treadmill and cycle EE ($P < 0.05$)
**Porto et al. (2009) (16)**	Cross-sectional study<br><br>(Comparison of Short-Term Heart Rate Variability: Wrist Monitor vs. ECG)	-n: 33<br>-15 males, 18 females<br>-Age: 18-42 years<br>-25 healthy subjects<br>-8 subjects with some isolated or associated abnormal manifestation that could potentially influence heart rate variability	EKG 12 lead	Polar S810 monito	Heart rate	-The Polar S810 monitor was feasible and reliable for recording short-term R-R interval series.<br>-It showed excellent agreement with the ECG for time-domain heart interval variability indexes.<br>-Differences between the two were functionally not relevant.<br>-The CV showed higher agreement in both postures, while SDNN and pNN50 had better agreement in the standing posture.
**Brugniaux et al. (2010) (24)**	Cross-sectional study<br><br>(assessment of energy expenditure with Polar Watch 200)	-n: 31<br>-15 males, 16 females<br>-Age: 33-35 yr<br>-Healthy population	Indirect calorimetry	Polar activity watch 200	- Energy expenditure	-EE values were identical for both methods during the first 90 minutes.<br>-At 120 minutes and the end of the hike, AW200 showed lower EE ($p < 0.05$).<br>-AW200 generally underestimates EE compared to Oxycon Mobil.
**Esliger et al. (2011) (45)**	Cross-sectional study<br><br>(validation of the GENEA accelerometer)	-n: 60<br>-Age: 40-63 years<br>-Healthy population	Actigraph, trixial RT3 accelerometer	GENEA accelerometer	Validity, value calibration	-GENEA showed high reliability with intrainstrument and interinstrument coefficients of variation of 1.8% and 2.4%.<br>-It demonstrated excellent criterion validity compared to the MAST acceleration ($r = 0.97$).

						-GENEA had high criterion validity (r = 0.85) with METs across wear positions in 10-12 static and dynamic physical activities.<br>-Criterion validity of GENEA was nearly identical to that of ActiGraph and RT3.
**Zhang et al. (2012) (46)**	Cross-sectional study<br><br>(classification of physical activity using GENEA)	-n: 60<br>-23 male, 37 female<br>-Age: 48.9 years ± 6.8<br>-Healthy population	-	GENEA accelerometer	Activity classification	-High correct classification rate based on streaming data features.<br>-Right wrist GENEA data accuracy: 96.97%, κ = 0.954.<br>-Left wrist GENEA data accuracy: 95.93%, κ = 0.934.<br>-10-fold cross-validation accuracy: 97.2% for right wrist, 96.01% for left wrist.
**Mannini et al. (2013) (32)**	Cross-sectional study<br><br>(classification of activity using a single accelerometer)	-n: 33<br>-11 males, 22 females<br>-Age 18-75 years<br>-Healthy population	-	Wockets are small, thin, and lightweight devices (43mm × 30 mm × 7 mm, 13g) that include a triaxial accelerometer (MMA7331LCR1), a microprocessor (ATMEGA1284P), a Bluetooth radio (RN-41) and a rechargeable Lithium battery (3.7V 240mAh)	Activity type and intensity estimation	-With 12.8 s windows, the ankle data achieved a high classification accuracy of 95.0%.<br>-Wrist data accuracy was 84.7%.<br>-Using 4 s windows, wrist data accuracy slightly decreased to 84.2%.
**Welch et al. (2013) (31)**	Cross-sectional study<br><br>(classification of accuracy of GENEA)	-n: 130<br>-48.5% males, 51.5% females<br>-Age: 41.2 years ± 10.9<br>-Healthy population	Wrist-worn, uniaxial accelerometer	GENEA accelerometer	Intensity categories	-Overall intensity classification accuracy: 52.9% across 14 activities<br>-Accurate classification (80%+): 5 activities (basketball, jogging, computer work, vacuuming, and walking on a treadmill)<br>-Accuracy for intensity levels:<br>• Sedentary: 69.76%<br>• Light: 44.86%<br>• Moderate: 46.2%<br>• Vigorous: 77.71%<br>-Basketball: accuracy > 80%<br>-Cycling activities: accuracy < 25% (expected due to activity type and GENEA placement)
**Tully et al. (2014) (27)**	Cross-sectional and validation study<br><br>(validation of Fitbit Zip^{TM})	-n: 47<br>-22 males, 25 females<br>-Age: 43 years<br>-Healthy population	ActiGraph GT3X accelerometer (Actigraph Inc, USA) and a Yamax CW700 pedometer (Yamax Inc, Japan)	Fitbit Zip^{TM}	Step count	-High correlation in steps/day between Fitbit Zip and reference devices.<br>-No significant difference between Fitbit and Yamax steps/day.<br>- Fitbit measured significantly more steps/day than Actigraph
**Takacs et al. (2014) (28)**	Cross-sectional and validation study	-n: 30<br>-15 males, 15 females<br>-Age: 29.6 years ± 5.7	Step count output was compared to observer counts and distance	FitbitOne	Step count and distance	-No significant differences between Fitbit One step count and observer counts, with substantial concordance (0.97-1.00)

	(validation of Fitbit One)	-Healthy population	output was compared to the calibrated treadmill output			-High inter-device reliability for step count across all walking speeds (ICC ≥ 0.95)<br>-Percent relative error for step count: less than 1.3%<br>-Significant difference in Fitbit One distance output vs. criterion values at all speeds ($P < 0.001$), with poor concordance (0.0-0.05)<br>-Excellent inter-device reliability for treadmill speeds (ICC ≥ 0.90)<br>-High percent relative error for distance (up to 39.6%)
**Storm et al. (2015) (12)**	Cross-sectional study<br><br>(detection and accuracy of steps in 7 activity monitors)	-n: 16<br>-10 males, 6 females<br>-Age: 28.87 years ± 2.65<br>-Healthy population	Two wireless inertial measurement units (OPAL, ADPM Inc., Portland, OR, USA)	Movemonitor (Mc Roberts), Up (Jawbone), One (Fitbit), ActivPAL (PAL Technologies Ltd.), Nike+ Fuelband (Nike Inc.), Tractivity (Kineteks Corp.) and Sensewear Armband Mini (Bodymedia)	Step count	-Step underestimation for devices (mean ± SD):<br>• Movemonitor: 15±33 steps<br>• One: 15±35 steps<br>• ActivPAL: 29±20 steps<br>• Up: 16±135 steps<br>• Tractivity: 36±178 steps<br>• Nike+ Fuelband: 253±331 steps<br>• Sensewear Armband Mini: 77±127 steps<br>-Movemonitor, One, ActivPAL, Nike+ Fuelband, and Sensewear Armband Mini underestimated steps at all walking speeds.<br>-Tractivity significantly overestimated step count.<br>-Movemonitor had the best performance, with <2% error at all speeds and the smallest error in outdoor walking.<br>-The Movemonitor excelled in walking recognition but struggled to distinguish between standing and sitting.
**El-Amrawy et al. (2015) (17)**	Cross-sectional study<br><br>(precision of wearable devices for Heart Rate (HR))	- n:4<br>-4 males<br>-Age: 26.5<br>-Healthy population	An observer counted steps using a tally counter, Onyx Vantage 9590 professional clinical pulse oximeter	The Apple Watch, Samsung Gear Fit, Samsung Gear 1, Samsung Gear 2, Samsung Gear S, iHealth Tracker (AM3), Pebble Steel, Pebble Watch, Qualcomm Toq, Motorola Moto 360, Garmin Vivofit, Mi Band, MisFit Shine, Jawbone Up, Nike+ Fuelband SE, Sony Smartwatch (SWR10), and FitBit Flex	Step count and heart rate	-Step accuracy ranged from 99.1% (MisFit Shine) to 79.8% (Samsung Gear 2), with precision from 4% (MisFit Shine) to 17.5% (Jawbone UP).<br>-Apple Watch had 99.1% accuracy for 200 steps, increasing to 99.5% for 1,000 steps, with the highest precision (CV = 2.6).<br>-MisFit Shine also showed high accuracy (99.7% for 1,000 steps).<br>-Pulse accuracy ranged from 99.9% (Apple Watch) to 92.8% (Moto 360), with precision from 5.9% (Apple Watch) to 20.6% (Samsung Gear S).

**Wallen et al. (2016) (18)**	Cross-sectional study<br><br>(accuracy of wearable devices for Heart Rate (HR))	- n:22<br>-11 males, 11 females<br>-Age: 24 years ± 5.6<br>-Healthy population	Electrocardiography (heart rate) and indirect calorimetry (energy expenditure)	Apple Watch (Apple Inc., California, United States), Fitbit Charge HR (Fitbit Inc., San Francisco, United States), Samsung Gear S (Samsung Electronics Co., Ltd., Suwon, South Korea) and Mio Alpha (Mio Global, Canada)	Heart rate, Energy expenditure	-Heart rate was more accurate than energy expenditure across all devices.<br>-Devices underestimated heart rate by 1–9% and energy expenditure by 9–43%.<br>-Samsung Gear S had the most variability in heart rate, Mio ALPHA in energy expenditure.<br>-Fitbit Charge HR and Mio ALPHA showed systematic bias.<br>-Apple Watch showed a significant skin type effect on heart rate correlation.<br>-Step count correlations were moderate to strong for Apple Watch (0.70), Fitbit Charge HR (0.67), and Samsung Gear S (0.88).<br>-Fitbit Charge HR had the most variability in steps, with a 4–6% underestimation.
**Roos et al. (2017) (8)**	Cross-sectional and validation study<br><br>(validity of watches estimating energy expenditure)	- n:20<br>-12 males, 8 females<br>-Age: 23.90 years ± 1.92<br>-Healthy population	Indirect calorimetry	Suunto Ambit2, Garmin Forerunner920XT, and Polar V800	Energy expenditure	-Aerobic running (4–11 km/h) errors: −25.16% to +38.09%.<br>-Polar V800 was most accurate (−12.20% to −4.29%).<br>-Garmin Forerunner 920XT underestimated (−25.16%) at slowest stage.<br>-Suunto Ambit2 overestimated (38.09% and 36.29%) at slower speeds.<br>-Anaerobic running (14–17 km/h): All devices underestimated by −21.62% to −49.30%.
**Shcherbina et al. (2017) (7)**	Cross-sectional study<br><br>(accuracy of wrist-worn sensors of heart rate and energy expenditure)	- n:60<br>-29 males, 31 females<br>-Age: ≥18 years<br>-Healthy population	Continuous 12-lead electrocardiographic (ECG) and indirect calorimetry	The first phase included the Apple Watch, Basis Peak, Fitbit Surge and Microsoft Band. The second phase included the MIO Alpha 2, PulseOn and Samsung Gear S2	Heart rate, energy expenditure	-Apple Watch had the lowest HR error (2.0%), while Samsung Gear S2 had the highest HR error (6.8%).<br>-EE estimation error was higher than HR for all devices.<br>-Lowest EE error: walking (31.8%) and running (31.0%), highest on sitting tasks (52.4%).<br>-No device had EE error below 20%.<br>-Errors were higher for males, greater BMI, darker skin tone, and walking.<br>-Apple Watch had the lowest error in both HR and EE; Samsung Gear S2 had the highest.
**Fokkema et al. (2017) (47)**	Cross-sectional and validation study<br><br>(Reliability and Validity of Ten Consumer Activity Trackers Depend on Walking Speed)	- n:31<br>-16 males, 15 females<br>-Age: 32 ± 12 years<br>-Healthy population	Hand counting	Polar Loop, Garmin Vivosmart, Fitbit Charge HR, Apple Watch Sport, Pebble Smartwatch, Samsung Gear S, Misfit Flash, Jawbone Up Move, Flyfit, and Moves	Step count	-Accuracy varied by tracker and walking speed, with differences ranging from 0.0% to 26.4% compared to the gold standard.<br>-The **Garmin Vivosmart** and **Fitbit Charge HR** were most accurate at slow walking speeds.<br>-The **Garmin Vivosmart** and **Apple Watch Sport** were most accurate at average walking speeds.<br>-The **Apple Watch Sport**, **Pebble Smartwatch**, and **Samsung Gear S** were most accurate at vigorous walking speeds
**Dooley et al. (2017) (14)**	Cross-sectional and validation study<br><br>(accuracy of heart rate and physical activity)	- n:31<br>-16 males, 15 females<br>-Age: 32 ± 12 years<br>-Healthy population	HR chest strap and a metabolic cart	Apple Watch, Fitbit Charge HR, and Garmin Forerunner 225.	Heart rate, energy expenditure	**-Apple Watch:** HR showed low variability with MAPE between 1.14% and 6.70%, but recorded lower HR during light, moderate intensity, and recovery stages. EE had a much higher MAPE (14.07% to

						210.84%) and consistently overestimated energy expenditure (EE) at all stages.<br>**-Fitbit:** HR had a MAPE between 2.38% and 16.99%, with higher HR readings during light intensity but lower during baseline, vigorous intensity, and recovery. EE had a MAPE between 16.85% and 84.98%, with higher readings at baseline, light, and moderate intensities.<br>**-Garmin Forerunner 225:** HR MAPE ranged from 7.87% to 24.38%, with higher readings across most stages. EE had a MAPE between 30.77% and 155.05%, with higher readings at all stages.<br>All devices generally overestimated energy expenditure, while HR accuracy varied with specific intensities, showing lower readings at lighter intensities for the Apple Watch and Fitbit, and higher readings for the Garmin
**Chowdhury et al. (2017) (48)**	Cross-sectional study<br><br>(assessment of energy expenditure)	- n:30<br>-15 males, 15 females<br>-Age: 27 ± 6 years<br>-Healthy population	Jawbone UP24 and validated research-grade multi-sensor devices (BodyMedia Core and individually calibrated Actiheart™)	Microsoft Band, Apple Watch (1) and Fitbit Charge HR	Energy expenditure	**-Apple Watch**: Best performance, lowest error, tightest limits of agreement for daily energy expenditure in both lab and free-living conditions.<br>**-Fitbit Charge HR**: Reasonable accuracy for free-living expenditure, but less consistent for specific activities in the lab.<br>**-Microsoft Band**: High error and bias, wider limits of agreement, consistent under-prediction in both lab and free-living conditions.<br>**-Jawbone UP24**: Higher error and bias, wider limits of agreement.<br>**-Sensewear**: Only device equivalent to Actiheart for energy expenditure estimates, meeting the ±10% equivalence zone.<br>**-None of the consumer devices** met the ±10% equivalence zone compared to Actiheart.
**Toth et al. (2018) (3)**	Cross-sectional and validation study<br><br>(assessment of step count)	- n:12<br>-6 males, 6 females<br>-Age: 35 ± 13 years<br>-Healthy population	GoPro videos were downloaded to a computer and researchers counted steps using a hand tally device	StepWatch (SW), activPAL, Fitbit Zip, Yamax Digi-Walker SW-200, New Lifestyles NL-2000,ActiGraph GT9X (AG),Fitbit Charge	Step count	**-SW devices** recorded 95.3% to 102.8% of actual steps ($P > 0.05$).<br>**-Fitbit Zip**, **Yamax Digi-Walker SW-200**, and **AG with the moving average vector magnitude algorithm on both wrists** recorded 71% to 91% of steps ($P > 0.05$).<br>**-ActivPAL**, **New Lifestyles NL-2000**, and **AG (no-LFE, moving average vector magnitude) worn on the hip**, and **Fitbit Charge** recorded 69% to 84% of steps ($P < 0.05$).<br>**-AG (no-LFE)** on both wrists recorded 109% to 122% of steps ($P > 0.05$).<br>**-AG (LFE)** on both wrists and the hip recorded 128% to 220% of steps ($P < 0.05$).

**Bai et al. (2018) (2)**	Cross-sectional study<br><br>(comparison of heart rate monitors)	- n:39<br>-16 males, 23 females<br>-Age: 19-60 years old<br>-Healthy population	Oxycon Mobile 5.0, Yamax SW-200 DigiWalker (DW) pedometer, Polar H7 HR sensor	Apple Watch 1 and Fitbit Charge HR.	energy expenditure (EE), steps, and heart rate	**-Apple Watch 1** was more accurate than **Fitbit Charge HR** for overall EE (15.2% vs. 32.9% MAPE).<br><br>-Both devices were accurate in **steps during aerobic activity**, but **Apple Watch 1** performed slightly better (6.2% vs. 9.4% MAPE).<br><br>**-Fitbit Charge HR** was most accurate for **heart rate** during sedentary behavior (7.2% MAPE) and comparable to **Polar monitor**.<br><br>**-Apple Watch 1** showed stronger validity for **EE** and **step tracking** during aerobic exercise.<br><br>-**Apple Watch 1** was better for EE and steps, while **Fitbit Charge HR** excelled in heart rate.
**Abt et al. (2018) (49)**	Cross-sectional study<br><br>(measurement of physical activity with Apple watch)	- n:20<br>-10 males, 10 females<br>-Age: 18-50 years old<br>-Healthy population	Breath-by-breath online gas analysis system (Cortex Metalyzer 3B, GmbH, Germany)	Apple watch	Oxygen consumption	-Large underestimation **of** moderate-intensity exercise **was observed when compared to the criterion of** 40% VO2R**.**<br><br>Significant **underestimation** of moderate-intensity exercise for both hands.
**Degroote el al. (2018) (50)**	Cross-sectional and validation study<br><br>(Accuracy and validation of smart devices)	- n:36<br>-18 males, 18 females<br>-Age: 20-65 years old<br>-Healthy population	The ActiGraph GT3X+	Fitbit Charge, Polar M600, Huawei Watch, and Asus Zenwatch 3	Steps and moderate to vigorous physical activity (MVPA)	-All 4 devices (**Huawei, Polar, Asus, Fitbit**) are valid for estimating **steps** in natural settings.<br><br>-For **MVPA**, all devices showed **systematic bias**, with **moderate to low validity** for both day and 15-min levels**.**
**Veerabhadrappa et al. (2018) (29)**	Cross-sectional and validation study<br><br>(Accuracy and validation of smart devices)	- n:71<br>-24 males, 47 females<br>-Age: 18-55 years old<br>-Healthy population	Video recorded using a Canon VIXIA HF500 camcorder placed perpendicular to the treadmill to observe the steps	Apple watch	Step count	**-Total error** for Apple Watch: 0.034% (1.07 steps) across all stages and participants.<br>- **Overestimated** step counts at slow (-2.6%), moderate (-0.9%), and brisk (-1.6%) paces.<br>**−Underestimated** step counts at jogging pace (+3.4%).<br>**−Least error** at moderate intensity (3.0 mph) with -0.9% relative error.<br>-No significant differences in step counts by gender, age group, or BMI.
**Xie et al. (2018) (19)**	Cross-sectional study<br><br>(comparing validity among different devices)	- n:44<br>-23 males, 21 females<br>-Age: 19-27 years old<br>-Healthy population	Manual measurements of the heart rate, number of steps, distance, and sleep, and energy	6 devices (Apple Watch 2, Samsung Gear S3, Jawbone Up3, Fitbit Surge, Huawei Talk Band B3,	Five major health indicators (heart rate, number of steps, distance, energy consumption, and	**−High accuracy** for heart rate, steps, distance, and sleep duration (MAPE ≈ 0.10).<br>**−Poor accuracy** for energy consumption (calories) with MAPE up to 0.44.

			consumption through oxygen consumption	and Xiaomi Mi Band 2) and 2 smartphone apps (Dongdong and Ledongli)	sleep duration) under various activity states (resting, walking, running, cycling, and sleeping)	-**Step measurement variation** was highest for **Apple Watch 2** (0.42) and lowest for **Dongdong** (0.01).<br>-**Heart rate measurement variation** was highest for **Samsung Gear S3** (0.34) and lowest for **Xiaomi Mi Band 2**(0.12).<br>-**Samsung Gear S3** was best for heart rate measurement at rest (MAPE 0.04).<br>-**Dongdong** was best for step measurement during walking (MAPE 0.01).<br>-**Fitbit Surge** was best for distance measurement during cycling (MAPE 0.04).<br>-**Huawei Talk Band B3** was best for energy consumption measurement during walking (MAPE 0.17).
**Boudreaux et al. (2018) (13)**	Cross-sectional and validation study<br><br>(comparing validity among different devices)	- n:50<br>-28 males, 22 females<br>-Age: 18-35 years old<br>-Healthy population	ECG,metabolic analyzer	Apple Watch Series 2, Fitbit Blaze, Fitbit Charge 2, Polar H7, Polar A360, Garmin Vivosmart HR, TomTom Touch, and Bose SoundSport Pulse (BSP) headphones.	Heart rate, energy expenditure	-**Polar H7 and BSP**: Valid for both exercise modes (Cycling: MAPE = 6.87%, **R** = 0.79; Resistance: MAPE = 6.31%, **R**= 0.83).<br>-**Apple Watch Series 2**: Best HR validity during cycling (MAPE = 4.14%, **R** = 0.80).<br>-**BSP**: Best HR accuracy during resistance exercise (MAPE = 6.24%, **R** = 0.86).<br>-**HR underestimation** increased with exercise intensity across all devices.<br>-No device valid for **EE** during cycling or resistance exercise.
**Gilgen-Ammann et al. (2019) (20)**	Cross-sectional study<br><br>(evaluation of quality of a heart rate monitor)	- n:10<br>-5 males, 5 females<br>-Age: 24.7 ± 1.9 years old<br>-Healthy population	ECG	Polar H10 heart rate monitor, medilog® AR12plus Holter monitor	Heart rate intervals	-**Medilog® AR12plus**: 94.6% RR interval signal quality.<br>-**Polar H10**: 99.6% RR interval signal quality, maintained 99.4% during high-intensity.<br>-High correlation between both (r = 0.997, p > 0.001).<br>-Both systems valid for RR interval detection across various activity intensities.<br>-**Polar H10** recommended as gold standard for intense activities.
**Zhang et al. (2020) (5)**	Cross-sectional study<br><br>(comparison of energy expenditure among wrist worn devices**)**	- n:30<br>-13 males, 17 females<br>-Age: 20.1 ± 1.7 years old<br>-Healthy population	Indirect calorimetry (metabolic cart (TrueOne 2400, Parvo Medics Inc., Sandy, UT, USA))	Apple Series 1 Watch, LifeTrak Core C200, and Fitbit Charge HR	Treadmill Energy Expenditure	-**Apple Watch**: Good agreement with indirect calorimetry at 160.9 m·min−1 (Mean diff = -2.7 kcals, MAPE = 11.9%) and 187.7 m·min−1 (Mean diff = 3.7 kcals, MAPE = 10.7%).<br>-**Fitbit**: Moderate agreement at 187.7 m·min−1 (Mean diff = -0.2 kcals, MAPE = 20.1%).<br>-**LifeTrak**: No statistical equivalence at any speed.<br>-**Apple Series 1** had narrower Bland-Altman limits, especially at slower speeds.<br>-Results support **Apple Watch** and **Fitbit Charge HR** for assessing energy expenditure during treadmill running speeds in young adults.
**Pope et al. (2019) (51)**	Cross-sectional and validation study	- n:21<br>-14 males, 7 females<br>-Age: 18-35 years old	ActiGraph GT3X+-BT accelerometers with an associated chest-	Apple Watch (AW), Fitbit Surge HR (FS), TomTom Multisport	Energy expenditure (EE), average heart rate (HR), and peak	-**ActiGraph** had moderate agreement with **FS** and **TT EE** (r = 0.62-0.69) and **HR** (r = 0.47-0.74).

	(Validation of Four Smartwatches in Energy Expenditure and Heart Rate**)**	-Healthy population	mounted ActiGraph HR strap (the Polar H7 Bluetooth HR monitor)	Cardio Watch (TT), and Microsoft Band (MB)	HR assessment during exergaming	-Differences found in **EE** ($P < 0.01$) and **HR** ($P < 0.01$) vs. **ActiGraph**.<br>-**Smartwatches** had low bias/adequate precision.<br>-**HR** measurements were moderately valid, but **EE** measurements were less valid.
**Pasadyn et al. (2019) (52)**	Cross-sectional study<br><br>(Accuracy of heart rate monitors**)**	- n: 30<br>-15 males, 15 females<br>- Age: 23.5 ± 3.0 years old<br>-Healthy population	Electrocardiogram (ECG)	Apple Watch, Fitbit Charge HR 2	Heart rate across different exercise intensities	-**Apple Watch** showed lower **RER** (2.4-5.1%) compared to **Fitbit** (3.9-13.5%) across all exercise intensities.<br>-Both devices had the strongest **ECG HR correlation** at very light physical activity (high CCC > 0.90).<br>-**Accuracy of HR monitoring** declined as exercise intensity increased for both **Apple Watch** and **Fitbit Charge HR2**.
**Sanudo et al. (2019) (53)**	Cross-sectional study<br><br>(Accuracy of heart rate monitors**)**	- n: 43<br>- 43 males<br>- Age: 20-43 years old<br>-Healthy population	Polar monitor	Apple watch	Heart rate	**-Device correlations** were very good (0.96-0.99, 95% CI: 0.94 to 0.99).<br>-**Skin type differences** (II vs. III) appeared with increased exercise intensity, but with trivial to small effect sizes (ES: 0.05 to 0.28).<br>-**Relative differences** between devices were <2%.<br>-**Apple Watch** showed trivial, systematic **underestimation of HR** compared to **Polar** across all skin types during exercise
**Nuss et al. (2019) (54)**	Cross-sectional observational study<br><br>(Accuracy of energy expenditure among smart watches**)**	- n: 30<br>-15 males, 15 females<br>- Age: 23.5 ± 2.95 years old<br>-Healthy population	PARVO metabolic cart (MetCart)	Fitbit Charge HR 2 (Fitbit) and the Apple Watch	Energy expenditure	-**Apple Watch** overestimated **EE** for women and underestimated **EE** for men.<br>-**Fitbit** consistently underestimated **EE** for both genders.
**Breteler et al. (2019) (30)**	Cross-sectional and validation study<br><br>(Validation of smart watches in step counts**)**	- n: 30<br>-14 males, 16 females<br>- Age: 23-58 years old<br>-Healthy population	Actigraph GT3X	Apple Watch, Misfit Shine, and iHealth Edge, Yamax Digiwalker (pedometer)	Step count in free-living conditions	-**Apple Watch** had the smallest **MAD** of 7.7% with the **Actigraph**.<br>-**Yamax Digiwalker** had the highest **MAD** of 20.3%.<br>-**iHealth Edge** and **Misfit Shine** had acceptable **MAD** values of 10.7% and 11.3%, respectively
**Nelson et al. (2019) (55)**	Cross-sectional study<br><br>(Accuracy of heart rate among smart watches)	- n: 1<br>-1 males<br>- Age: 29 years old<br>-Healthy population	Ambulatory ECG (Vrije Universiteit Ambulatory Monitoring System)	Apple Watch 3 and Fitbit Charge 2	Heart rate	-A**pple Watch 3**: Mean difference of -1.80 bpm, MAE of 5.86%, and 95% agreement with ECG over 24 hours.<br>-**Fitbit Charge 2**: Mean difference of -3.47 bpm, MAE of 5.96%, and 91% agreement with ECG over 24 hours.<br>-Both devices showed high accuracy across the 24-hour condition.
**Speer et al. (2020) (56)**	Cross-sectional study<br><br>(Accuracy of heart rate among smart watches in young children**)**	- n: 19<br>-7 males, 12 females<br>- Age: 4 ± 0.58 years old<br>-Healthy population	Electrocardiogram (ECG)	Finger photoplethysmography (PPG), Polar H10 heart rate (HR) sensor	Heart rate (RR intervals)	-No **HRV** parameters were valid for the **PPG** system.<br>-**Polar H10** showed moderate to good relative reliability (ICC = 0.50–0.90) for **R-R intervals** and **HR variability**.

						-**Polar H10** displayed better absolute reliability with **TEM% < 12%** for **R-R intervals**, **HR**, and **RMSSD**.<br>-**Polar H10** is recommended for **HRV measurement** in young children in real-world educational settings.
**Duking et al. (2020) (57)**	Validation study<br><br>(validity of heart rate and energy expenditure wrist-worn wearables**)**	- n: 25<br>- 11 males, 14 females<br>- Age: 26 ± 7 years old<br>-Healthy population	Polar H7, indirect calorimetry	Apple Watch 4, Polar Vintage V, Garmin Fenix 5, Fitbit Versa	Heart rate, Energy expenditure	-**Apple Watch**: Highest **HR accuracy** with absolute errors of ~3.9 bpm deviation.<br>-**Apple Watch** & **Polar Vantage V**: Less influenced by exercise intensity compared to **Fitbit** & **Garmin**.<br>-**Apple Watch**: Highest **EE accuracy** with **MAPE** = 15.2%.
**Chow et al. (2020) (58)**	Validation study<br><br>(Accuracy of Optical Heart Rate Sensing Technology in Wearable Fitness Trackers**)**	- n: 40<br>-20 males, 20 females<br>- Age:<br>20 younger adults (20-26) & 20 older adults (65+)<br>-Healthy population	Polar H7	Xiaomi Mi Band 2 and Garmin Vivosmart HR+	Heart rate	-**MAPE values** were below 10% for both devices.<br>-**Bland-Altman analysis** showed both devices **underestimated** the average heart rate.
**Prim et al. (2021) (59)**	Cross-sectional study<br><br>(Assessment of heart rate among smart watches in military population**)**	- n: 15 patients<br>- 13 males, 2 females<br>- Age: 18-45 years old<br>-Healthy population	Electrocardiogram (ECG)	Faros 180 ECG monitor (Mega Electronics Ltd.) and Polar H10 monitor (Polar Electro Oy).	Heart rate (heart rate variability HRV)	-The **Faros 180** was less accurate in detecting **HR peaks** during pushups than the **Polar H10**, requiring more than 5% editing of total IBIs.<br>-**Polar H10** was reliable compared to the **gold-standard ECG**.
**Henriksen et al. (2022) (60)**	Validation study<br><br>(Validation and comparison study among new smart devices**)**	- n: 21 patients<br>- 57% males, 43% females<br>- Age: 33 ± 14 years old<br>-Healthy population	Actigraph accelerometer, Actiheart accelerometer	Polar Vantage, Oura Ring	Sleep, RHR, EE	-**Oura** and **Polar** had poor **steps** accuracy with MAPEs of 69% and 72%, respectively.<br>-**Oura** and **Polar** also had poor **EE** accuracy with MAPEs of 13% and 19%, respectively.
**Miller et al. (2022) (34)**	Validation study<br><br>(Validation of wrist-worn devices for the measurement of heart rate and energy expenditure**)**	- n:53 patients<br>- 27 males, 26 females<br>- Age: 25 ± 3 years old<br>-Healthy population	Polar H10, GPS tracker model etc.	APPLE Watch S6, GARMIN Forerunner 245, POLAR Vantage V, OURA Ring 2, WHOOP 3.0, SOMFIT	Energy expenditure	-**Heart rate accuracy**: Ranged between 2% and 7%.<br>-**Energy expenditure**: Ranged between 10% and 35%, with accuracy decreasing as intensity increased.<br>-**Apple Watch**: Tended to overestimate heart rate with a 3% error.
**Hajj-Boutros et al. (2023) (4)**	Randomized controlled trial<br><br>(Validation of wrist-worn devices for the measurement of heart rate and energy expenditure**)**	- n:60 patients<br>- 30 males, 30 females<br>- Age: 25 ± 3 years old<br>-Healthy population	Polar H10, Metamax 3B	APPLE Watch 6, POLAR Vantage V, FITBIT Sense	Energy expenditure, heart rate	-**Apple Watch**: Highest **HR** accuracy with <5%.<br>-**Energy expenditure (EE)** accuracy: Poor, with a range of 13% to 30%
**Giordano et al. (2025) (21)**	Experimental validation and feasibility	- n:10 young adults<br>-Healthy population	ECG, Motion artifact testing	Wrist-worn Internet of Things (IoT) device with ultrasound for	Heart rate	-**High correlation** with ECG: Heart rate extracted from ultrasound signals showed a **Pearson correlation coefficient of r(92) = 0.99, p < 0.001.**

	(Develop and validate a low-power ultrasound wrist device for accurate, robust, and continuous heart rate monitoring in wearable applications)			low-power, high-fidelity heart rate extraction		
**Heinz et al. (2025) (25)**	Experimental<br><br>(Evaluate the accuracy of the Apple Watch Series 7 in estimating energy expenditure during EMS-supported exercise compared o spirometric calorimetry.	- n: 35 young adults<br>- Mean age of 26.8 ± 6.2 years (range: 18-40)<br>- 18 males, 17 females<br>-Healthy population	Spirometric calorimetry measured with the MetaLyzer 3B (CORTEX Biophysik GmbH, Leipzig, Germany)	Apple Watch Series 7 (Model A2478, watchOS Version 9.6.3)	Energy expenditure	-The **Apple Watch Series 7 underestimated EE** compared to **spirometric calorimetry** – overall **mean difference = -27.4 kcal; limits of agreement = 62.2 kcal.**<br>-Strong correlations between the **Apple Watch and spirometry**: **r = 0.93** for all data. Similar correlation (**r = 0.93**) during exercise with and without electromyostimulation (EMS).<br>-The **Apple Watch** showed consistent underestimation during exercise without EMS – **mean difference -28.8 kcal** – and with EMS – **mean difference -26.0 kcal.**
**Lopez-Ortiz et al. (2025) (22)**	Cross-sectional validation<br><br>(Validate the accuracy and reliability of the low-cost KCO8 smartwatch for measuring blood pressure, heart rate, and blood oxygen saturation compared to reference devices)	- n: 100 Caucasian adults<br>-Mean age of 41 ± 18 years<br>-Participants with and without diseases, but excluded if difference of at least 20 mm Hg in their systolic and/or diastolic BP between their two arms.	Omron M7 automatic cuff-based upper-arm sphygmomanometer	KCO8 smartwatch (low-cost wearable device equipped with optical sensors intended to measure blood pressure, blood oxygen saturation (SpO_2), and heart rate	Primary: systolic and diastolic blood pressure<br>Secondary: heart rate and blood oxygen saturation	-**Blood pressure**: There are significant differences between the **KCO8** and **reference** for systolic and diastolic blood pressure (**p < 0.05**). **KCO8** showed **low intra-subject variability (CV: 1.6% for systolic, 1.9% for diastolic). Poor reliability** indicated by **very low ICC values (ICC ≈ 0.054 for systolic, ≈ -0.027 for diastolic).** No significant correlation between **KCO8** and **reference** BP measurements.<br>-**Heart rate**: No significant differences in **mean HR** between **KCO8** and **reference (p = 0.123)**. **Strong correlation** between **KCO8** and **reference HR (r ≈ 0.815**). ICC indicated **excellent reliability (ICC ≈ 0.898**).<br>-**SpO_2:** Significant difference between **KCO8** and **reference** (**p < 0.001**). **Very low ICC** (**≈ 0.059**), indicating **poor reliability.** No significant correlation between devices (**r ≈ 0.039**).
**Oropesa et al. (2025) (23)**	Experimental and cross-sectional validation<br><br>(Evaluate the validity of 16 PPG-based wearable heart rate monitors across different exercise intensities and protocols, including high-intensity interval training)	- n: 10 well-trained male endurance athletes and triathletes<br>-Mean age = 27 years<br>-With extensive training experience and high aerobic capacity<br>-Healthy population	Polar H9 chest strap	Amazfit Fit5<br>Apple Ultra and Apple SE<br>Garmin models: 35, 45, 235, 935<br>Polar models: M200, M430, OH1 (can be worn on the wrist), Vantage M, Vantage V, Vantage V3, Verity Sense<br>Galaxy Watch 5<br>Suunto Fitness	Heart rate	-**All devices** showed **excellent overall agreement (ICC > 0.90) during the full exercise protocols.**<br>-**Accuracy** declined at higher exercise intensities (**> 160 bpm)** and during **HIIT**, with increased **bias, SEM, and CV.**<br>-**Apple devices** (e.g., Apple Ultra) exhibited the **highest validity, minimal errors, and no data loss.**<br>-Some devices (e.g., **Garmin 35, Polar M200**) experienced significant **data loss** and required data refinement, especially during HIIT.<br>-**Data loss and measurement errors** were more pronounced at **high intensities** and during **rapid HR fluctuations**.

**Rosenbach et al. (2025) (61)**	Validation<br><br>(Validate Garmin's stress score against physiological measures and predict self-reported stress using wearable devices during controlled mental stress tasks.	- n:60 healthy university students<br>-Mean age = 27.5 years (range 19-39)<br>-20 males, 40 females<br>-Healthy population	ECG recordings derived from Polar H10 chest strap	Garmin Vivosmart 4	Heart rate<br>Heart rate variability (HRV) metrics	-High correlation with **HR (r ≈ 0.84)** between **Garmin Vivosmart 4** and the **Polar H10 ECG**.<br>-Moderate correlation with **HRV metrics** between **Garmin** and **Polar H10 ECG.**
**Zhao et al. (2025) (26)**	Validation<br><br>(Evaluate the accuracy of popular smartwatches in estimating energy expenditure during aerobic dance protocols: HIIT, MICT, and TABATA)	- n: 20 athletes<br>-10 males, 10 females<br>-Mean age: 21.10 ± 1.59<br>-Healthy population	Indirect calorimety (MetaMax 3B) and Scott's method Actigraph	Apple Watch Ultra<br>Huawei Watch 4<br>Amazfit Balance<br>Samsung Galaxy Watch 6	Energy expenditure	-**ActiGraph accelerometers** showed the highest accuracy with an **average MAPE of 14.51%**<br>-All devices tested consistently **underestimated EE**, with **MAPE ranging from 16.6% to 28.4%** across different protocols.<br>-The **Huawei Watch 4** showed substantial **overestimation with MAPE reaching up to 42.8%** under higher-intensity activity.
